# Evaluation of *in vivo* digital root reconstruction based on anatomical characteristics of the periodontal ligament using cone beam computed tomography

**DOI:** 10.1038/s41598-017-18592-4

**Published:** 2018-01-10

**Authors:** Chenxin Wang, Yi Liu, Siwei Wang, Yong Wang, Yijiao Zhao

**Affiliations:** 1 Center of Digital Dentistry, Peking University School and Hospital of Stomatology, Beijing Shi, China; 2 Department of Orthodontics, Peking University School and Hospital of Stomatology, Beijing Shi, China; 3 National Engineering Laboratory for Digital and Material Technology of Stomatology, Beijing Shi, China; 4 Research Center of Engineering and Technology for Digital Dentistry of Ministry of Health, Beijing Shi, China; 5 Beijing Key Laboratory of Digital Stomatology, Beijing Shi, China

## Abstract

This study’s aim was to develop and validate an approach to automatically extract and reconstruct three-dimensional (3D) digital root models from *in vivo* teeth based on the anatomical characteristics of the periodontal ligament using cone beam computed tomography (CBCT) data. Prior to undergoing dental extractions for orthodontic purposes, the CBCT data of each study participant were collected and imported into Mimics software to reconstruct 3D *in vivo* digital root models (test models). Twenty roots of 17 teeth extracted from the study’s participants were scanned using a dental scanner to obtain 3D *in vitro* digital root models (reference models). The 3D morphological deviation between the reference and test models was compared; the 3D size of the bucco-lingual, mesio-distal, and root length dimensions were calculated. This approach achieved an average 3D morphological deviation of 0.21 mm, and the average size error in the bucco-lingual, mesio-distal, and root length dimensions were −0.35 mm, −0.17 mm, and 0.47 mm, respectively. This new automatic extraction approach rapidly and accurately reconstructs 3D *in vivo* root models with detailed morphological information, and has the potential to improve diagnostic and treatment work flow in orthodontic clinics, as well as in other areas of dentistry.

## Introduction

With the development of digital dentistry, cone beam computed tomography (CBCT) has been widely used in the diagnosis and analysis of oral diseases^1–3^, especially when combined with image segmentation and three-dimensional (3D) reconstruction technologies in digital software, which make 3D measurements of *in vivo* teeth possible^4–6^. Having knowledge of the patients’ individualized, *in vivo* 3D tooth root morphology is important for dentists, especially orthodontists^7^; tooth arrangement^8^ and root information provided by 3D virtual reality techniques can assist in the diagnosis of disease and design of treatment plans more accurately and precisely^9,10^. Additionally, having accurate 3D morphological information regarding *in vivo* roots is also essential for computer-aided design/computer-aided manufacturing (CAD/CAM), individualized root-shaped implants^11^, periodontal surgery^12^, and complex root canal therapy^13^.

The 3D shape of a tooth’s crown can be easily obtained using the digital impression technology from intra- or extra-oral devices. However, the 3D morphology of the *in vivo* root can presently only be obtained using sequence tomography of the root first, then indirectly obtaining the 3D reconstruction using greyscale segmentation in professional medical imaging software^14^. During this process, the segmentation of the root’s contour from sequential greyscale CBCT images is the most important step. However, accurate extraction of the 3D digital root model *in vivo* is challenging due to the unique characteristics of the tooth’s structure. First, the density gradient of the tooth changes from the crown to the root, which subsequently induces changes in the root’s greyscale in the sequential CBCT images. This makes it impossible to extract and segment the information with a uniform threshold range in the software. Second, the greyscale of the physiological images of dentine and alveolar bone are very similar, and the structures are physically adjacent to one another. This creates a problem in that the boundary between the root and alveolar bone on greyscale images is difficult to identify clearly; therefore, it is difficult for the existing imaging software that is based on greyscale difference segmentation to achieve automatic and precise extraction^15^.

Previous studies have proposed two ways to solve these root image segmentation issues. The first uses manual segmentation to modify the greyscale images of the root layer-by-layer according to the dentist’s subjective experience^14,16^. The advantage of this method is that it ensures the accuracy of the data extraction, but its drawbacks are the intensive workload and low extraction efficiency. The second method involves the development of algorithms be used in proprietary software. Existing algorithms primarily include the region growing algorithm^17^, triangle mesh model algorithm^18^, mean shift algorithm^19^, and level set segmentation algorithm^20^. These algorithms are based on professional software programming, and are rarely used in the clinical setting.

The objective of this study was to explore and evaluate a new approach for rapidly reconstructing a 3D digital root model of an *in vivo* tooth from large field-of-view CBCT data. This approach is based on the anatomical characteristics of the periodontal ligament, with the combined application of Morphology and Boolean Operations, and Smart Expand mathematical functions in Mimics software, an advanced medical imaging software with reliable algorithms and functions being used in oral clinics. This approach can achieve fast and automatic root differentiation from alveolar bone with very few human interventions, and has the advantage of high automation and efficiency, as well as acceptable accuracy.

## Materials and Methods

This study was approved by the bioethics committee of the Peking University School and Hospital of Stomatology (PKUSSIRB-201631112) and was carried out in accordance with approved guidelines for research involving human subjects. The procedures and risks involved with the participation in this study were discussed with the volunteers, and written informed consent was obtained from each participant.

### Experimental equipment and settings

Large field-of-view CBCT (NewTom VGi, Italy) imaging was performed with 0.3 mm-voxel resolution according to the practice standards of the Department of Orthodontics, Peking University School and Hospital of Stomatology. The scanning parameters were as follows: tube voltage, 110 V; tube current, 2 mA; and exposure time, 10 s. The large field-of-view CBCT imaging include acquisition of 3D images of the craniofacial hard and soft tissues, alveolar bone, and teeth. DICOM data were imported into Mimics 19.0 software (Materialise, Belgium) and *in vivo* root digital models were segmented and reconstructed. After tooth extraction in the clinic, the extracted teeth were scanned by a structured-light 3D scanner (Smart Optics 880, Germany) with an accuracy of 0.02 mm to obtain *in vitro* root digital models, which served as reference models. Both the *in vivo* and *in vitro* root models were imported into measurement software (Geomagic Studio 2012, 3D System, USA), and the 3D accuracy of the root reconstructions was evaluated by the function of 3D error analysis. An image processing software (Adobe Photoshop CS3, California, USA) were used to deal with the figures.

### Experimental data acquisition

Ten patients (3 males and 7 females) from the orthodontics department who required dental extractions for orthodontic treatment participated in this study. After providing informed consent, all patients underwent NewTom VGi CBCT scanning with a large field-of-view in the conventional head position prior to tooth extraction. Seventeen complete teeth, including eight maxillary first premolars, three maxillary second premolars, one mandibular first premolar, two mandibular second premolars, two maxillary third molars, and one mandibular third molar, with 20 roots in total, were then extracted and collected for use in this study. All teeth were rinsed in running water immediately after extraction to remove residual blood, periodontal tissue, and calculus, and then stored in normal saline. After drying the extracted teeth, the 3D *in vitro* root digital models, including a portion of the crown, were obtained using the Smart Optics scanner, and then were exported in STL format for use as the reference models (Root Model_1).

### 3D reconstruction of the *in vivo* digital root model

**Figure 1:**
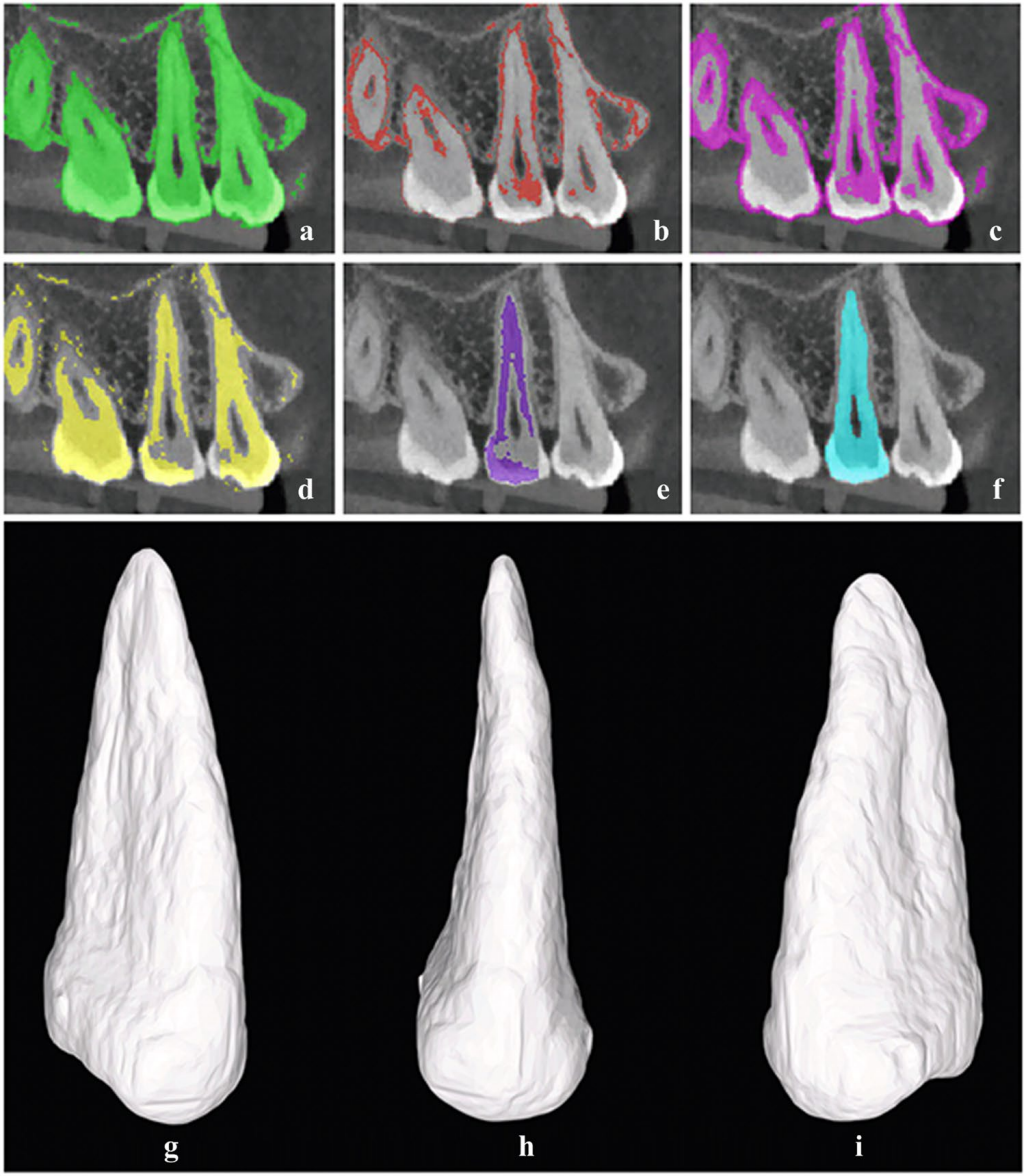
The segmentation and extraction of the digital *in vivo* root model in Mimics software and the 3D *in vivo* root digital model obtained after reconstruction. (**a**) The root-bone mask. (**b**) The periodontal ligament mask. (**c**) The extended periodontal ligament mask. (**d**) The root seed mask. (**e**) The root seed mask of the individual tooth. (**f**) The extended individual seed mask. (**g**) Buccal-Distal view. (**h**) Buccal view. (**i**) Buccal-Mesial view.

The patients’ CBCT data in DICOM format were imported into Mimics 19.0 software. Segmentation was performed using the threshold function to set up an appropriate threshold range for converting the greyscale images of the soft and hard tissue of the extraction area. This ensured that the crown, root, peripheral alveolar bone (primarily cortical bone), and periodontal ligament were included in the mask area, i.e., the “root-bone mask” (Fig. 1a). The threshold range was then adjusted to isolate the periodontal ligament, and a new threshold mask, known as the “periodontal ligament mask” (Fig. 1b), was created. In the CBCT images obtained using a low radiation dose, the periodontal ligament mask demonstrated discontinuous distribution.

The “26-connectivity extension” function in the Morphology Operations tool was applied to extend the periodontal ligament mask by 1–2 voxels, extending the discontinuous distribution into a continuous distribution, which we termed the “extended periodontal ligament mask” (Fig. 1c). The extended periodontal ligament mask was subtracted from the root-bone mask by application of Boolean Operations to obtain a region of interest we termed the “root seed mask” (Fig. 1d), which is the key to successful root segmentation. A small number of manual edits were needed to correct the following issues: 1. The adjacent teeth (primarily the coronal portion) in the mask were disconnected; 2. The remaining contact region with the alveolar bone was disconnected; and 3. The border of the root seed mask was slightly less than the border of the root in the image. Precise contour trimming was not required to edit the root seed mask.

After the root seed mask was manually edited, the Region Growing tool was applied to separate the independent seed regions of the corresponding teeth (the “individual root seed mask”; Fig. 1e). The Smart Expand function, which is a boundary identification function based on a grey-gradient identification algorithm, was applied to perform controllable 3D voxel expansion of the individual root seed mask with a configurable parameter, the “maximum step size”; this ensured that the scope of the mask extended from the root seed region to the entire tooth area (Fig. 1F).

After 3D reconstruction, the 3D *in vivo* digital root model was exported in STL format to serve as the test model, which we named Root Model_2. The data from 20 roots in total were processed using this approach.

### Evaluation of reconstruction accuracy

**Figure 2:**
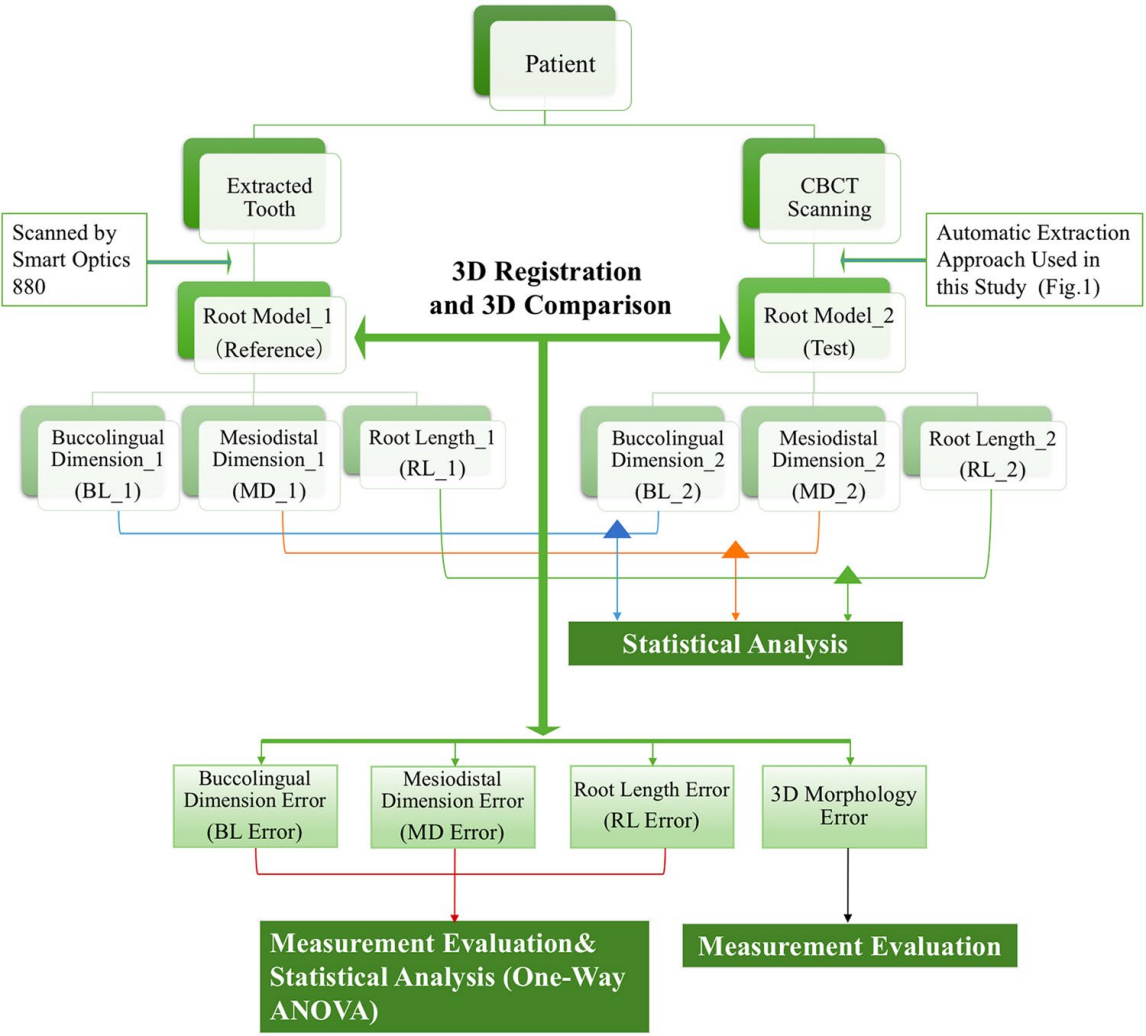
Workflow of the experimental procedures and evaluation methods.

The evaluation of the accuracy of the reconstructions was divided into measurement evaluation and statistical analysis (Fig. 2).

#### Measurement evaluation

The Root Model_1 and Root Model_2 data (in STL format) for the same tooth were imported into Geomagic Studio 2012 software. The Registration function was used to align the two models based on their common region of interest on the root. The 3D curve of the cementoenamel junction was extracted from Root Model_1 and projected onto Root Model_2. The two models were then cut with the same junction, leaving only the root portion remaining.

Root Model_1 was used as the reference model and Root Model_2 was used as the test model for application of the Deviation Analysis function in Geomagic Studio. The differences in 3D morphology between the two models was calculated using the Root Mean Square (RMS) parameter and was displayed as different coloured images to intuitively represent the overall 3D morphological error. Equation 1 defines the RMS value.

1 $$RMS=\sqrt{\frac{{\sum }_{{\rm{i}}=1}^{N}{X}_{{\rm{i}}}^{2}}{N}}=\sqrt{\frac{{X}_{1}^{2}+{X}_{2}^{2}+\ldots +{X}_{N}^{2}}{N}}$$

The value of Xi represents the spatial linear distance between the nearest corresponding point of the reference and test models. And the value of N represents the number of pairs of points on the two models involved in the deviation calculation. The average value of N was 6,000–10,000 in this study. In particular, the approximate number of points and faces in Root Model_1 are 13383 and 26300, in Root Model_2 are 7241 and 14291(after cutting the cementoenamel).

**Figure 3:**
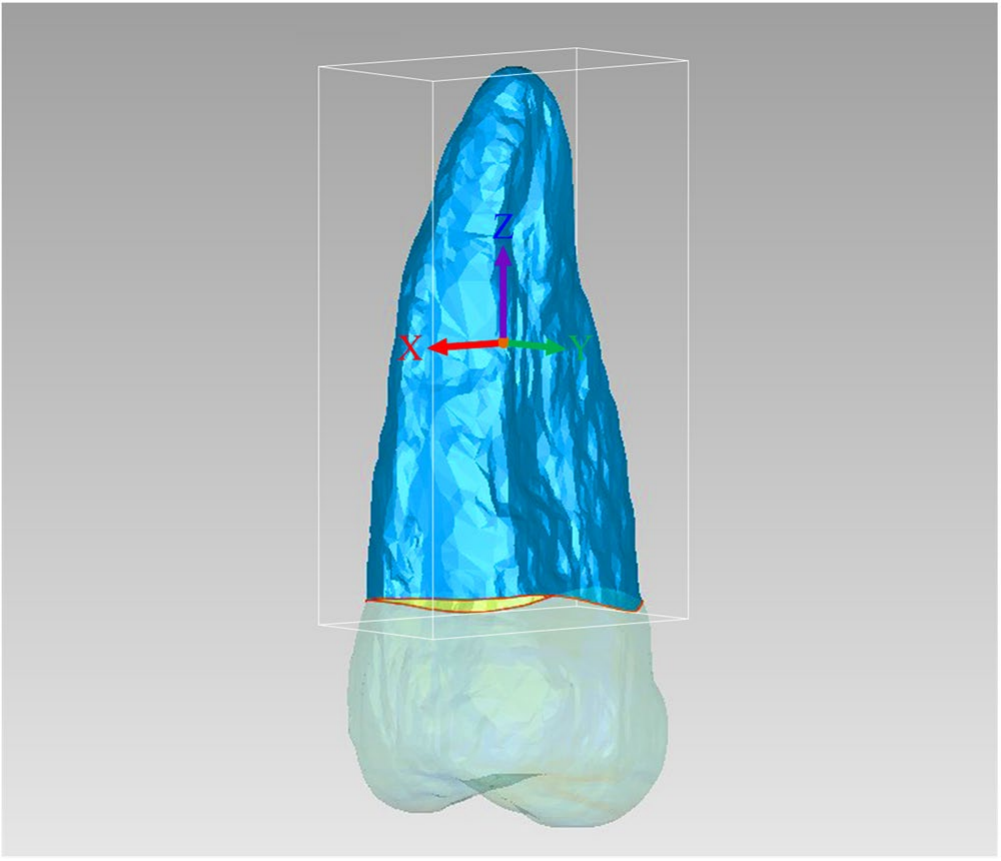
The local coordinate system of the 3D root model. X-axis (red): bucco-lingual direction. Y-axis (green): mesio-distal direction. Z-axis (purple): root length direction. White border: maximum bounding box.

The model coordinate system for each tooth was established in the Geomagic Studio software, in which the bucco-lingual, mesio-distal, and root length directions (Fig. 3) were represented by the X-axis, Y-axis, and Z-axis, respectively. The maximum size of the root model in all three directions of the coordinate system was calculated using the Maximum Bounding Box function. The error between Root Model_1 and Root Model_2 for the bucco-lingual, mesio-distal, and root length dimensions were also calculated by subtracting Root Model_2 from Root Model_1 to evaluate the accuracy of our reconstruction approach:

1. Bucco-lingual dimension error (BL Error) = Bucco-lingual dimension of Root Model_1 (BL_1) − Bucco-lingual dimension of Root Model_2 (BL_2).
2. Mesio-distal dimension error (MD Error) = Mesio-distal dimension of Root Model_1 (MD_1)− Mesio-distal dimension of Root Model_2 (MD_2).
3. Root length error (RL Error) = Root length of Root Model_1 (RL_1) − Root length of Root Model_2 (RL_2).

#### Statistical analysis

All statistical analyses were performed with SPSS (Version 19, SPSS Inc., Chicago, IL, USA). A K-S normality test was conducted for BL_1, MD_1, RL_1, BL_2, MD_2, and RL_2 to examine the distribution of the data (20 calculated values for each group). A parametric or non-parametric test was then used depending on the results. One-way ANOVA was performed for the BL, MD, and RL errors. The Bonferroni post-hoc test was used to perform multiple comparisons. The statistical significance was set at P < 0.05.

### Data availability

The data sets generated and analysed during the current study are available from the corresponding author upon reasonable request.

## Results

### 3D morphological error

**Figure 4:**
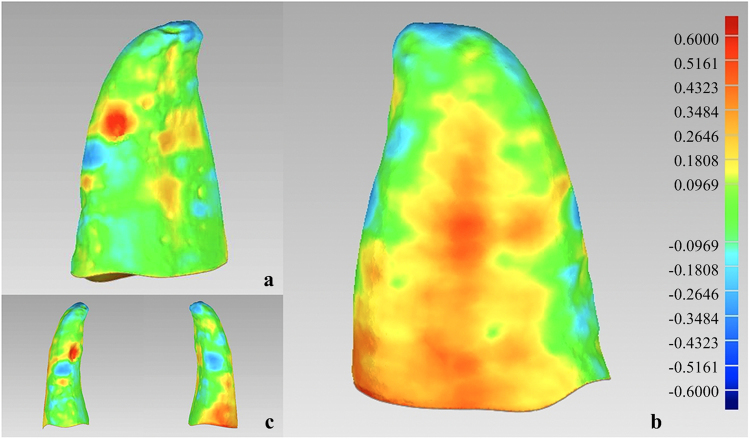
Different coloured images representing different levels of error between Root Model_2 and Root Model_1. The maximum error range was set between −0.6 and +0.6 mm. The areas of positive error are represented by the yellow and red regions, and the areas of negative error are represented by the blue regions. Areas where the error is near zero are represented by the green regions. (**a**) Distal view. (**b**) Mesial view. (**c**) Buccal and lingual views.

Utilization of different coloured images (Fig. 4) allowed qualitative congruency analysis between the test (Root Model_2) and reference (Root Model_1) models. The maximum error range was set between −0.6 and + 0.6 mm. The areas of positive error are represented by the yellow and red regions, and the areas of negative error are represented by the blue regions. Areas where the error is near zero are represented by the green regions. The mean ± standard deviation (SD) of the RMS values are 0.21 ± 0.05 mm, representing the overall level of 3D morphological error, which could meet the orthodontic accuracy requirements for clinical root extraction and reconstruction^8,14,21^.

### 3D root size error

**Figure 5:**
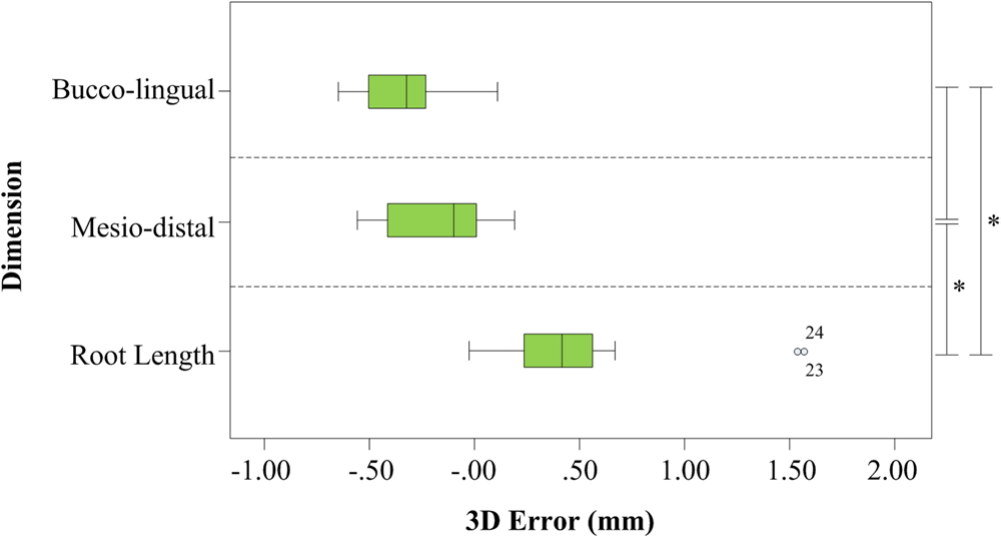
Boxplot of the 3D error measurements for the bucco-lingual, mesio-distal, and root length dimensions. The circles within the boxplot represent outliers, and the asterisks on the right sidebar signify P < 0.05.

Table 1 reports the measurement values of the bucco-lingual, mesio-distal, and root length dimensions for the 20 roots of 17 teeth, and the 3D size errors of the BL, MD, and RL errors. The mean ± SD of the BL, MD, and RL errors were −0.35 ± 0.18 mm, −0.17 ± 0.25 mm, and 0.47 ± 0.41 mm, respectively. The average RL errors of single-rooted and double-rooted teeth were 0.42 mm and 0.56 mm, respectively. The results of the K-S normality test showed that six data sets were non-normally distributed, thus two groups of related non-parametric Wilcoxon signed-rank tests were performed between BL_1 and BL_2, MD_1 and MD_2, and RL_1 and RL_2. Table 2 shows that the two models had statistically significant differences (P < 0.05) on all three tests. Table 3 shows the result of the one-way ANOVA among the BL, MD, and RL errors. Homogeneity of variance testing revealed an equal variance for the three data groups (P > 0.05). Bonferroni post-hoc testing revealed that the RL error was significantly different from the BL and MD errors (Fig. 5).

**Table 1:** Measurement values of the bucco-lingual and mesio-distal dimensions, and root length for the 20 roots of 17 teeth and the 3D size errors.

Items	Bucco-lingual Dimension (mm)			Mesio-distal Dimension (mm)			Root Length (mm)		
No.	BL_1	BL_2	BL Error	MD_1	MD_2	MD Error	RL_1	RL_2	RL Error
1	9.57	9.95	−0.38	5.82	6.15	−0.33	16.13	14.59	1.54
2	10.4	10.79	−0.39	7.09	7.5	−0.41	9.31	7.74	1.57
3	9.46	9.8	−0.34	9.06	9.55	−0.49	8.96	8.39	0.57
4	9.05	9.54	−0.49	5.37	5.94	−0.57	11.98	12.01	−0.03
5	8.86	9.38	−0.52	5.43	5.84	−0.41	12.32	11.76	0.56
6	8.3	8.77	−0.47	5.01	4.94	0.07	12.07	11.62	0.45
7	8.2	8.84	−0.64	4.65	5.09	−0.44	13.3	13.15	0.15
8	9.2	9.41	−0.21	5.68	5.53	0.15	13.98	13.85	0.13
9	9.9	10.07	−0.17	6.1	6.11	−0.01	12.85	12.27	0.58
10	9.59	9.84	−0.25	5.86	5.88	−0.02	13.34	13.26	0.08
11	10.23	10.88	−0.65	6.04	6.52	−0.48	8.47	7.8	0.67
12	8.81	9.32	−0.51	5.59	5.58	0.01	13.99	13.64	0.35
13	8.53	8.83	−0.3	5.38	5.44	−0.06	15.16	14.83	0.33
14	8.63	8.87	−0.24	5.58	5.39	0.19	15.21	14.81	0.4
15	7.16	7.06	0.1	5.67	6.07	−0.4	13.72	13.48	0.24
16	8.81	9.32	−0.51	5.59	5.58	0.01	12.08	11.63	0.45
17	8.53	8.83	−0.3	5.38	5.44	−0.06	14.15	13.71	0.44
18	8.63	8.87	−0.24	5.58	5.39	0.19	14.19	13.73	0.46
19	8.02	8.31	−0.29	5.29	5.43	−0.14	11.51	11.15	0.36
20	9.1	9.31	−0.21	5.88	6.03	−0.15	9.57	9.33	0.24

Abbreviations: BL_1, bucco-lingual dimension of Root Model_1; BL_2, bucco-lingual dimension of Root Model_2; MD_1, mesio-distal dimension of Root Model_1; MD_2, mesio-distal dimension of Root Model_2; RL_1, root length of Root Model_1; RL_2, root length of Root Model_2; BL Error, bucco-lingual dimension error; MD Error, mesio-distal dimension error; RL Error, root length error.

**Table 2:** Results of the Wilcoxon signed-rank test comparing BL_1 and BL_2, MD_1 and MD_2, RL_1 and RL_2. Abbreviations: BL_1, bucco-lingual dimension of Root Model_1; BL_2, bucco-lingual dimension of Root Model_2; MD_1, mesio-distal dimension of Root Model_1; MD_2, mesio-distal dimension of Root Model_2; RL_1, root length of Root Model_1; RL_2, root length of Root Model_2. ^*^P < 0.05.

Paired Sample Group	P value of Wilcoxon signed-rank test
BL_1 and BL_2	<0.05^*^
MD_1 and MD_2	<0.05^*^
RL_1 and RL_2	<0.05^*^

**Table 3:** Results of the one-way ANOVA for BL, MD, and RL errors. Abbreviation: BL Error, bucco-lingual dimension error; MD Error, mesio-distal dimension error; RL Error, root length error. ^*^P < 0.05.

Multiple Comparisons (Bonferroni)		
*Dimension*	*Dimension*	*Significance*
BL error	MD error	0.172
	RL error	8.94 × 10^−12*^
MD error	BL error	0.172
	RL error	1.49 × 10^−8*^
RL error	MD error	1.49 × 10^−8*^
	BL error	8.94 × 10^−12*^

## Discussion

The X-ray absorption of the cranial-maxillofacial tissues was recorded through the greyscale information in the tomographic images. To a certain extent, this recording method reflects the density of human tissues, but it cannot distinguish different organizational structures with similar density. Low-dose radiation CBCT is commonly used in oral clinics and image information for the upper and lower jaw, including the tooth crowns and roots, and alveolar bone can be obtained under large field resolution^22^. The ability of the dentist to manually edit the greyscale images layer-by-layer has become an indispensable method for the extraction of *in vivo* root information; however, the workload of completing this task for the entire dentition, which is required for digital orthodontic diagnosis, is very extensive.

The periodontal ligament is a 0.15–0.38-mm thick, fibrous connective tissue structure that separates the tooth root from the alveolar bone^23^. On large field-of-view CBCT images, the periodontal ligament appears as an extremely narrow, low-brightness/shadowy area between the root and alveolar bone. Because the physical size of the periodontal ligament is smaller than the voxel resolution of the CBCT image under the large field-of-view (0.3 mm), the image of the periodontal ligament generally appears as an intermittently striped shadow around the root. Therefore, the periodontal ligament cannot be used to completely separate the root from the alveolar bone using the medical imaging software (like Mimics). Despite the lack of continuity of the periodontal ligament on CBCT images, its 3D spatial distribution still represents the 3D morphological features of the root to a certain degree. Therefore, in this study, we used the Morphology Expansion algorithm to expand the discontinuous periodontal ligament image by 1 or 2 voxel units in order to achieve 3D transfixion. Though the periodontal ligament mask after expansion is thicker than the actual periodontal ligament, it retains its 3D spatial distribution, allowing the root and alveolar bone to be automatically separated using the periodontal ligament mask. Requiring less manual editing in the software, the contour features of the root region can be completely separated from the alveolar bone using the automatic segmentation function. After the interference of the alveolar bone image is removed, the Smart Expand function, based on the greyscale gradient recognition algorithm, can easily be used to expand the boundary of the “seed region” to the real root-bone boundary. Therefore, fast and efficient root segmentation and extraction can be achieved. According to our preliminary statistics, under the circumstances of same operating conditions, it takes approximately 8 to 10 minutes to extract a single rooted tooth model using the conventional manual segmentation method and 15 to 20 minutes for double rooted tooth model. Applying our approach, the average time for the extraction and reconstruction of 20 roots is approximately 4 to 5 minutes. Statistical analysis of extraction efficiency needs to be studied further.

As demonstrated in our results, the P-values of the Wilcoxon signed-ranks test between the reference and test models for the bucco-lingual, mesio-distal, and root length dimensions were less than 0.05, indicating a statistically significant difference between the reference and test models. One possible reason for these results may be that the scanning accuracy of CBCT (0.3 mm) in the test group was much lower than that of the Smart Optics (0.02 mm) system used in the reference group^24^. In 2010, Liu *et al*.^14^ extracted *in vivo* tooth information using manual threshold segmentation in editing software and compared this with the *in vitro* tooth volume of the control group. The results of the analysis revealed that there was a statistically significant difference between the traditional manual segmentation and control groups. The authors believed that the quality of CBCT is an important factor affecting the extraction accuracy, which is in agreement with the results of our study. It can be seen that the accuracy of the extraction method based on large field-of-view CBCT is significantly different from that of *in vitro* tooth scanning, whether utilizing manual segmentation or the automatic extraction approach based on the periodontal ligament that we used in this study. Therefore, improving the scanning accuracy of CBCT may effectively improve the extraction accuracy of this approach^24^. The performance of *in vivo* root model extraction from high resolution CBCT data needs to be further investigated^25^. For example, one approach might be to use different manufacturers and different resolution CBCT to extract and reconstruct the *in vivo* root model, to compare the accuracy between them. Meanwhile, it is necessary to compare the proposed approach with manual and automatic segmentation in order to determine the efficiency of the reconstruction.

The mean ± SD of the bucco-lingual, mesio-distal, and root length dimension errors are −0.35 ± 0.18 mm, −0.17 ± 0.25 mm, and 0.47 ± 0.41 mm, respectively for the 20 roots of the 17 teeth examined in this study. The results of the measurement evaluation demonstrate that Root Model_2 extracted using our approach tends to have a greater bucco-lingual and mesio-distal dimension (a negative mean), but a smaller root length dimension (a positive mean) compared to the reference model. Meanwhile, the results of the one-way ANOVA of the BL, MD, and RL errors show that the RL error was significantly different from the BL and MD errors. There was no statistically significant difference between the BL and MD errors. Combined with the above measurement results, we demonstrated that the errors of the bucco-lingual and mesio-distal dimensions of the 3D root digital model extracted by this approach are relatively acceptable, but the error of root length is slightly less acceptable.

The results obtained in this study are related to the parameters of two key steps in our approach. First, during the periodontal ligament expansion stage, the Morphological Operations tool provided by Mimics can extend the unit voxel by “8-connexity” or “26-connexity”. From our pre-experiment, we found that the 8-connexity extension algorithm (in slice plane) could not be adapted to all patients’ periodontal ligament images, while the 26-connexity spatial extension algorithm could be adapted to all patients in this study and obtain 3D continuity. However, the drawback is that the extended range is slightly larger, so the accuracy of the seed region range can be influenced to a certain degree after applying Boolean Operations. In addition, during the phase of smart expansion of the root seed mask, the maximum expansion step parameters provided by Mimics need to be evaluated comprehensively based on the greyscale gradient performance of the patient’s root region images and the extraction range of the seed mask, which often requires several attempts to determine the parameters. Second, in this study, the extracted 3D root models often required surface smoothing to improve visualization in the clinical application; however, some scholars have reported that surface smoothing reduces the size of the tooth model^14^. Therefore, this should be considered carefully when determining the range of parameters of the algorithm, which may lead to a larger discrepancy of the bucco-lingual and mesio-distal dimensions. In addition, more stringent parameter settings can be applied to the model to meet a clinician’s personalized needs.

Regarding the observation of shorter average root length, another study reported that there is a relationship between the shorter root phenomenon and the imaging accuracy of large field-of-view CBCT^24^. The NewTom VGi CBCT device used in this study has a voxel resolution of 0.3 mm in a large field-of-view, and the shooting dose is relatively small, so the apical region of the root cannot be clearly identified on CBCT images^15,26^. Owing to the 1–2 layers of informational loss on CBCT images of the root-tip area, the computer software algorithm is less accurate for the root length dimension than other two dimensions.

Additionally, the patient was in the natural head position during CBCT imaging for the orthodontic treatment^27^. For the physiologic angle of the premolars, the sequence images within the range of 30° along the long axis (the root length index measured in this study was along the customized long axis) of the tooth can be obtained, and the loss of root length was approximately 0.42 mm in single-rooted teeth and 0.56 mm in double-rooted teeth. This demonstrates that the extraction error of the root length is related to the imaging angle of the root on the CBCT images. The results of this study showed that the root length error of double-rooted teeth was greater than that of the single-rooted teeth, but a study with a larger sample size is needed for further statistical analysis.

Although an evaluation of full dentition segmentation was not included in this study, we believe that higher extraction efficiency can theoretically be achieved. For the same patient, the dental and periodontal tissue in different tooth positions have similar imaging performance, so the extraction process of the roots of the full dentition require less manual editing to modify the root-bone boundary and all teeth within the full dentition range could be extracted at once. With the help of 3D post-editing in the software for crown segmentation, theoretically, more efficient individual tooth model extraction could be achieved. Further research is required to verify these theories.

In conclusion, we have developed and validated an approach to automatically extract and reconstruct 3D *in vivo* root digital models based on the anatomical characteristics of the periodontal ligament. Compared with previously proposed methods, this technique can achieve automatic root differentiation from the alveolar bone with minimal human intervention, and has the advantages of being highly automated and efficient, as well as having acceptable accuracy. Possession of the 3D morphological information of the *in vivo* root provided by the reconstructed model can assist with orthodontic tooth arrangement, the development of individualized implants, periodontal surgery, and complex root canal therapy. Future studies will aim at exploring the performance of *in vivo* root model extraction from high resolution CBCT data, comparing this approach with manual and automatic segmentation for efficiency evaluation and determining if there is a significant difference in the root length error between single-rooted and double-rooted teeth. Based on our findings, it would also be valuable to investigate reconstruction of the full dentition, which may achieve higher extraction efficiency.
